# Metabolomics as a Powerful Tool for Molecular Quality Assessment of the Fish *Sparus aurata*

**DOI:** 10.3390/nu3020212

**Published:** 2011-02-11

**Authors:** Gianfranco Picone, Søren Balling Engelsen, Francesco Savorani, Silvia Testi, Anna Badiani, Francesco Capozzi

**Affiliations:** 1 Department of Food Science, University of Bologna, Piazza Goidanich 60, 47023 Cesena (FC), Italy; Email: gianfranco.picone2@unibo.it; 2 Quality & Technology, Department of Food Science, Faculty of Life Sciences, University of Copenhagen, Rolighedsvej 30, 1958 Frederiksberg C, Denmark; Email: se@life.ku.dk (S.B.E.); frsa@life.ku.dk (F.S.); 3 Aquaculture and Fisheries Science Laboratories, Alma Mater Studiorum, University of Bologna, Viale Vespucci 2, 47042 Cesenatico (FC), Italy; Email: silvia.testi@unibo.it; 4 Department of Veterinary Medical Sciences, Alma Mater Studiorum, University of Bologna, Via Tolara di Sopra 50, 40064 Ozzano Emilia (BO), Italy; Email: anna.badiani@unibo.it; 5 Centre of Magnetic Resonance, University of Florence, Via L. Sacconi 6, 50019 Sesto Fiorentino (FI), Italy

**Keywords:** gilthead sea bream, fish, quality assessment, aquaculture, metabolic profile, ^1^H-NMR, metabolomics, chemometrics

## Abstract

The molecular profiles of perchloric acid solutions extracted from the flesh of *Sparus aurata* fish specimens, produced according to different aquaculture systems, have been investigated. The ^1^H-NMR spectra of aqueous extracts are indicative of differences in the metabolite content of fish reared under different conditions that are already distinguishable at their capture, and substantially maintain the same differences in their molecular profiles after sixteen days of storage under ice. The fish metabolic profiles are studied by top-down chemometric analysis. The results of this exploratory investigation show that the fish metabolome accurately reflects the rearing conditions. The level of many metabolites co-vary with the rearing conditions and a few metabolites are quantified including glycogen (stress indicator), histidine, alanine and glycine which all display significant changes dependent on the aquaculture system and on the storage times.

## 1. Introduction

In the last decade, several scientific studies on fish and seafood, including wild and farmed fish and shellfish, both of marine and freshwater origin, have shed light on the importance of these products in human diet as healthy products [1,2,3]. The main quality aspect derives from the nutritional value due especially to the presence of essential amino acids (arginine, histidine, isoleucine, leucine, lysine, methionine, plenylalanine, threonine, tryptophan and valine), highly digestible proteins, vitamins (A, D and B complex), minerals, and a high content of polyunsaturated fatty acids (PUFA). The latter represents the only source of ω-3 fatty acids, whose importance in reducing the risk of certain human diseases is well established and abundantly presented in literature [4,5,6].

The importance of seafood has become so relevant that in less than fifty years the global demand for fish products has doubled and is still rising [7]. This aspect, together with the development of new technologies in fishing activities and industrial production through selective breeding, hybridization, and the application of biotechnology [7], has lead to a change from traditional fishing to fish farming.

Due to its increasing importance on the global economical scene, aquaculture has become an important subject which requires particular attention from both a safety and quality point of view. The quality of farmed fish depends not only on its intrinsic characteristics such as species, age and gender, but also on factors such as the developmental phase, environmental temperature, feed regime, capture method and composition of lipids in the diet [8]. In this way, the global quality of fish is also influenced by the harvesting and post-harvesting procedures (aquaculture) [9]. The definition of quality related to seafood, thus, becomes really complex. On the other hand, the fish quality desired by consumers is strictly related to its nutritional value, flavor and others sensory components [10] based on the chemical composition of flesh. It thus becomes of prime importance, to be able to evaluate this diversity of quality and to be able to compare the whole metabolic profile of fish muscle among different methods of farming.

The Gilthead sea bream (*Sparus aurata*) is an example of economical fish species whose market has rapidly increased in the last 20 years [11] and now is the object of an intensive farming practice all along the Mediterranean basin [12]. This intensive production, under “artificial” conditions, has raised problems with respect to the quality of the farmed fish compared to the wild one. These problems are magnified by the way this species is still predominantly stored, that is whole, under ice [13]. The ice storage is considered to be a useful way of prolonging the shelf life, preserving the original quality of seafood but, at the same time, may lead to the development of unacceptable features [14]. This method of storage makes the product very sensitive to its initial metabolic state which may compromise not only quality attributes for fresh consumption, but also the technological properties of fish during further industrial processing [15]. The loss of freshness and thus the shelf-life is strictly related to changes that take place early *post mortem*. These changes are due both to the modification of the structure and the biochemistry of the cells and to the proliferation of microorganisms [16]. 

From an analytical point of view, the estimation of freshness can be derived from the measurement of several indicators of fish proprieties [17]. In most cases, these properties are dependent on different biological and processing factors occurring *post mortem* that influence the degree of physical, chemical, biochemical and microbiological changes [18]. The evaluation of the modification of the status of freshness in fish fillet in *post mortem* phase can be derived from the quantification of the production of undesirable compounds, such as amines [19] (mainly trimethylamine [20]), other volatile bases [21] and nucleotides [22,23]. In the last case, nucleotides produced by the adenosine-5’-triphosphate (ATP) decomposition, such as adenosine-5’-diphosphate (ADP) and adenosine-5’-monophosphate (AMP), inosine (INO) and inosine-monophosphate (IMP), as well as hypoxantine (HX), are considered useful indicators of fish freshness [24,25], and their amount is combined to define the K-value parameter [22,26]. Nowadays, the use of the K-value as a universal method to assess fish quality related to freshness is still questionable because of individual variation between species due to sampling and to the different ratio of dark and white muscle. Moreover, the K-value is affected by the catch area, season, fishing gear and weight [27].

The work reported here is aimed at evaluating the changes, occurring in the whole molecular profile of aqueous extracts obtained from Gilthead sea bream fish, as a consequence of different aquaculture systems and of storage of the fish under ice. Scarce literature exists on the changes observed in the aqueous metabolome of Gilthead sea bream when stored, under ice, as a whole non eviscerated fish [28,29,30].

The comprehensive molecular composition, as observed by NMR spectroscopy, can provide an objective point of view to evaluate differences that may be reflected in the nutritional and sensory attributes of the fish. Nuclear magnetic resonance (NMR) spectroscopy is largely employed in metabolic analysis of tissue extracts because it generates a large quantity of informative spectral data in a short time. However, NMR spectra of biological samples are extremely complex and an optimized method to explore the information from the NMR spectral data is needed: this is made possible using multivariate data analysis which, in this context, is called chemometrics [31]. The fish metabolome studied under different rearing conditions is a perfect example of a self-organizing system and chemometrics respects the autonomy of nature in soft modeling of data through outlier validation that first makes induction measuring, and hypothesizing afterwards, a scientific process [32]. 

Principal component analysis (PCA) [33] is a commonly used chemometric tool as it simplifies the multivariate data into a few dimensions that can be readily understood and evaluated. In this manner, each sample (spectrum) can be represented by relatively few numbers (PC scores) instead of thousands of variables (spectral data points). PC scores can then be plotted, making it possible to visually assess similarities and differences between samples and to determine whether samples can be grouped into meaningful patterns. As an exploratory method, PCA is most commonly used to identify how one sample is different from another, and which variables contribute the most to this difference.

In this work, three different kinds of aquaculture practices have been considered, namely cage (CG), tank (TK) and lagoon (LG) environments. For each aquaculture, the effect of ice-storage time on the evolution of metabolites has been evaluated and samples captured, sacrificed and immediately stored under ice (T_0_), and then compared to samples stored under ice for sixteen days after sacrifice (T_16_).

## 2. Experimental Section

### 2.1. Sampling

To study the influence of the ice storage and aquaculture system on the whole metabolic profile, samples were taken from white muscle of caudal part of Gilthead sea bream harvested in three different aquaculture systems: tanks, cages and lagoons.

In the first case (tanks), all batches comprised three two years old individuals with a body weight between 300 and 400 g (*i.e.*, within the commercial size), fed with a commercial feed containing 46% proteins and 21% lipids, and harvested in October 2006. Before death, all fish fasted for 24 h. Before sample excision, all fish were kept under ice for 6 h, in order to consider the same starting time (T_0_) of *postmortem* for all samples, including those arriving from distant farms that required 4–6 h for transport.

With regards cages fish farming, gilthead sea breams were caught from sea cages in Monfalcone (Italy). Fish were two years old and the capture occurred in January 2007. Following a feeding consisting of a commercial feed (45% proteins and 16–18% lipids) and because of the low water temperature (averaging about 3 degrees), the fish had been fasting for two months, a common practice to prevent the winter disease, for which the combination of feeding and low temperatures is considered one of the main etiological factors.

Lagoons samples were represented by batches composed of three two-year-old individuals having commercial size. These fish were not fed a commercial feed, thus all nutrients came from natural environmental resources mainly characterized by benthos. This fish farming was developed at Valle Smarlacca S.r.l. in Ravenna (Italy) and placed in the Adriatic Sea.

From capture to killing and storage, all specimens underwent the same treatment and they were always kept in polystyrene boxes under ice flakes. The fish were taken to the laboratory in polystyrene boxes filled with ice flakes.

### 2.2. Experimental Design and Sample Preparation

For each of the three aquaculture systems, six fish were sampled. The white right caudal muscle (WRCM) was promptly cut out of three fish per aquaculture and immediately stored at −80 °C (T_0_ samples). The remaining specimens were left for 16 days under ice before taking tissue samples from the same muscle (T_16_ samples). Also these samples were stored at −80 °C prior to preparation for NMR analysis. 

A perchloric acid extraction was performed for each biological sample (*n* = 18) in triplicate. This setup yielded a total of 54 samples for each of which a high resolution NMR spectrum was recorded. For each sample, three aliquots of four grams of WRCM (replicates) were separately frozen with liquid nitrogen and then ground in a ceramic grinding vessel. Eight milliliters of 7% perchloric acid was added to the resulting powder and mixed until a perfect homogenization was reached. The acid mixtures, transferred into 2 mL centrifuge tubes, were neutralized to pH 7.8 using 9 M KOH and then centrifuged at 8000 rpm for 20 min at 4 °C in order to remove potassium perchlorate precipitate. The resulting supernatants from each sample were recombined and dispensed in 1.00 mL aliquots in Eppendorf tubes (1.8 mL) and stored at −80 °C until NMR analysis. All steps were performed in a cold room (4 °C). 

All chemical reagents used were of analytical grade and were purchased from SIGMA-ALDRICH Inc., St. Louis, MO, U.S.

### 2.3. NMR Data Acquisition and Processing

For each sample a FID was acquired for a total of 54 ^1^H-NMR spectra. Before the acquisition, a 10% (v/v) D_2_O was added to each sample for deuterium lock. pH was measured and eventually adjusted to 7.80, and then centrifuged at 14,000 rpm for 5 min at room temperature. A volume of 800 μL was transferred to a 5 mm NMR tube. The ^1^H-NMR spectra were recorded at *T* = 298 K on a Varian Mercury-plus spectrometer, operating at ^1^H frequency of 400 MHz, using a 5 mm triple resonance indirect detection probe; for each spectrum 1024 scans were acquired with a standard PRESAT pulse sequence, applied to allow an efficient water suppression. Data were collected over 32 K data points with a spectral width of 16 ppm, a pulse angle of 60°, a recycle delay of 2.0 s, and acquisition time of 2.561 s with a constant receiver gain. Before Fourier transformation, a line broadening of 1.60 HZ was applied to all Free Induction Decays (FID) and all final data spectra were referred to Creatine (3.04 ppm). MestReC 4.9.8.0 Software (Mestrelab Research SL, Santiago de Compostela, Spain) was used to phase and baseline correct all the spectra which were then converted in to ASCII.

### 2.4. Data Preprocessing and Multivariate Data Analysis

The spectra were first tidied by eliminating data points containing only the residual signal from the solvent (between 4.85 and 4.50 ppm) and those at both edges, which contain only noise (between 12.61 and 8.75 ppm, and between −0.55 and −3.38 ppm). The final reduced spectra were organized as a 54 × 18,000 matrix in which each row represents a sample and each column a spectral point. This matrix was pre-processed using home-made algorithms written in the R program language (The R Project for Statistical Computing, version 2.4.0.). A normalization preprocessing step was first applied on the entire matrix to minimize the differences due to the sample extract dilutions. For each row (spectrum), each column (variable) was divided by the sum of the absolute values of all variables, obtaining a vector with unit area. Before multivariate data analysis, the pre-processed matrix was condensed by subdividing each row into 150 bins, each integrating 120 data points (0.058 ppm). This practice is commonly used for high-field NMR data both for reducing the number of total variables employed making data mining faster and for correcting for small peak misalignment problems, mainly due to slight pH differences that can significantly affect the peak chemical shift of pH-dependent molecules.

Principal Component Analysis (PCA). To identify changes in metabolic profiles among samples in an unsupervised manner, principal components analysis of the preprocessed and mean-centered NMR data was performed using R.

## 3. Results and Discussion

**Figure 1:**
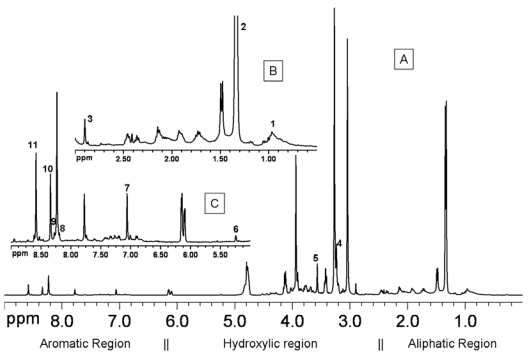
^1^H-NMR spectrum (**A**) of perchloric acid extracts of Gilthead sea bream fillet. The aliphatic (**B**) and the aromatic region (**C**) have been, respectively, 3× and 15× vertically expanded in order to show the presence of weaker signals. Signals selected for quantitative analysis have been labeled with numbers, according to Table 1.

The ^1^H-NMR spectroscopy, performed on perchloric acid solutions extracted from fish flesh, provides spectra constituted by a huge amount of data points tracing a molecular profile crowded by thousands of signals belonging to molecules containing at least one hydrogen nucleus (e.g., almost all biological compounds). Moreover, the position of signals in the spectra provides information on the chemical nature of the molecules in the sample. The spectrum of biological samples can, advantageously, be divided up into three main sub-regions, namely the aliphatic, the hydroxylic and the aromatic regions (Figure 1).

The aliphatic region is mainly occupied by signals belonging to organic acids, side-chains of amino acids, lipids (practically not present in the aqueous extracts) or, in general, hydrocarbon moieties of hydrophilic molecules which are not affected by nearby electron-attractor groups. The hydroxylic region contains signals belonging to alcohols and poly-alcohols such as sugars, but also to the alpha amino acids or to the unsaturated compounds. The aromatic region contains signals of (poly) phenols and other aromatics, including purine derivatives such as ATP and its catabolites.

NMR spectra permit to quantify changes occurring in the chemical composition of a mixture by following two different approaches: (1) by selecting specific metabolites, identifying their signals by spectral assignment, and observing the changes occurring in their area integrals along all the acquired spectra; (2) by identifying all signals undergoing changes in the area integrals along the whole spectral dataset, and assigning them to their corresponding molecules producing a list of metabolites as objects of study. The first approach is used in Table 1 where a few number of *a priori* identified metabolites are identified in the spectra and quantified, and where the outcomes of the Student’s paired *t*-test (*p* ≤ 0.05) used to compare T_0_ and T_16_ within parameters and aquaculture system are reported. By and large glycogen seems to have decreased significantly, which is in line with what was expected as a consequence of the *post mortem* glycolysis [34], although muscle lactate, probably due to the quite long storage period, could already have undergone depletion. An increase in TMA was predictable, in that, in marine fish it is derived from the breakdown of the osmoregulator trimethylamine oxide (TMAO) during spoilage [35]. *Post mortem* nucleotide degradation, which leads from ATP to hypoxanthine through a well known host of compounds [36], was behind the depletion of A(T,D,M)P and IMP, and the accumulation of inosine and hypoxanthine.

**Table 1:** Concentrations of main metabolites in Gilthead sea bream at time 0 and after storage under ice for 16 days.

T_0_								
			Tanks		Lagoons		Cages	
Metabolite		Reference signal ^a^ (ppm)	Conc. ^b^ (μmol g^−1^)	Sdev (±)	Conc. ^b^ (μmol g^−1^)	Sdev (±)	Conc. ^b^ (μmol g^−1^)	Sdev (±)
1	Leucine	0.96 (d)	0.19	0.04	0.10	0.01	0.084	0.01
2	Lactate	1.33 (d)	4.29	0.09	4.0	0.3	3.6	0.1
3	TMA	2.89 (s)	0.01	0.001	0.011	0.002	0.012	0.003
4	Taurine	3.24 (t)	0.43	0.02	0.39	0.07	0.86	0.02
5	Glycine	3.56 (d)	0.34	0.01	0.45	0.04	0.8	0.3
6	Glycogen	5.24 (s)	0.03	0.01	0.018	0.004	0.014	0.004
7	Histidine	7.05 (s)	0.38	0.03	0.24	0.03	0.29	0.04
8	Hypoxanthine	8.18 (s)	0.03	0.01	0.028	0.006	0.021	0.002
9	A(T,D,M)P	8.27 (s)	0.05	0.02	0.055	0.003	0.053	0.005
10	Inosine	8.34 (s)	0.02	0.01	0.036	0.007	0.039	0.005
11	Inosine 5-MP	8.57 (s)	0.52	0.04	0.49	0.04	0.4	0.1
**T_16_**								
1	Leucine	0.96 (d)	0.25	0.06	*0.15	0.03	*0.19	0.05
2	Lactate	1.33 (d)	*3.59	0.09	*3.6	0.1	3.64	0.06
3	TMA	2.89 (s)	0.013	0.003	*0.013	0.001	*0.017	0.001
4	Taurine	3.24 (t)	*0.55	0.09	*0.53	0.04	*0.7	0.2
5	Glycine	3.56 (d)	*0.27	0.02	*0.50	0.04	0.93	0.08
6	Glycogen	5.24 (s)	*0.02	0.01	0.02	0.01	*0.009	0.004
7	Histidine	7.05 (s)	*0.31	0.02	*0.22	0.01	*0.38	0.02
8	Hypoxanthine	8.18 (s)	0.03	0.01	*0.036	0.004	*0.033	0.003
9	A(T,D,M)P	8.27 (s)	0.046	0.003	*0.045	0.003	0.054	0.003
10	Inosine	8.34 (s)	*0.19	0.03	*0.233	0.007	*0.23	0.02
11	Inosine 5-MP	8.57 (s)	*0.29	0.02	*0.29	0.02	0.30	0.01

^a^ Chemical shift of the signal used for quantitative determination—Signal multiplicity (s, singlet; d, doublet; t, triplet); ^b^ Concentration; According to the Student *t*-test, metabolites showing statistically significant differences (*p* < 0.05) between T_0_ and T_16_ within the same aquaculture system have been indicated with *.

When scrutinizing the table with the metabolite quantification, some significant differences amongst different aquacultures are revealed but this approach also has its limitations. Only metabolites that are known and considered important are included and the analysis will thus confirm what was already known to be important, and the chances of obtaining new insight is therefore seriously hampered. It would be much more rewarding to combine the table with an exploratory investigation of the complex fish metabolome data hosted by the NMR spectra. This can be achieved by conducting a multivariate data analysis of the matrix of NMR spectral data. PCA is the most common approach for exploratory studies as it has the advantage to display the statistical variance of whole spectra among different groups of samples, by providing intra-group dispersions, not necessarily requiring delving into detail with the chemical composition of samples. PC scores plots and PC loadings plots graphically illustrate the results of PCA.

**Figure 2:**
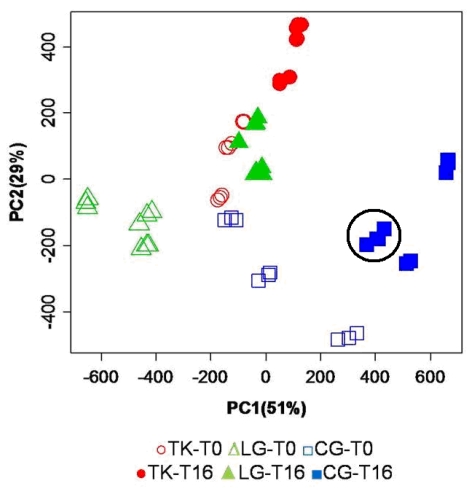
PC scores plot resulting from PCA of the mean centered binned spectral data set. Open symbols refer to samples immediately extracted after fish sacrifice, while filled symbols refer to samples extracted from fish stored under ice for 16 days. For each fish specimen, 3 different replicates have been measured, resulting in a nice and consistent grouping on the PC scores spaces as exemplified by the black circle.

The PC scores (PCs) plot, from the principal component analysis performed on the normalized and binned spectral data set, are shown in Figure 2.

In the scores plot in Figure 2, PC1 and PC2 explain 51% and 29%, respectively, of the total variance. The combination of the first two PCs is able to spontaneously cluster the samples according to the aquaculture practice. A clear separation is observed among the three aquaculture groups, both for fresh samples (open symbols) and for stored samples (filled symbols). A higher intra-group variance is observed for cage reared fish, indicating a larger heterogeneity of molecular profiles for this aquaculture system at T_0_. PC1 alone is able to clearly distinguish lagoon fish (LG-T_0_) from the other two groups at T_0_, as well as the fish reared in cages (CG-T_16_) from the other two groups at T_16_. On the other hand, PC2 alone is able to differentiate between fish reared in tanks (TK-T_16_) and those captured in lagoons or cages at T_16_.

PCA gives rise to some considerations in terms of general quality of fish, as affected by storage time and farming conditions. First of all, time evolution of metabolites profile shifts PC scores in a right-up direction independently from the aquaculture systems. The same dislocation is observed between lagoon and tanks groups at T_0_, as if the latter had the same metabolic composition of older samples as the first one. Conversely, the cage group at T_0_ is located in a position of the PC scores plot that may reflect differences in composition unrelated to metabolites involved in the time-dependent degradation. In summary, a combination of PC1 and PC2 describes a picture capable of capturing differences in the chemical composition of flesh aqueous extracts immediately after fish sacrifice. Furthermore, PCA indicates a similar time evolution of the metabolic profile of fish stored under ice, independent of their rearing conditions. This substantial similarity is reflected by the PC scores of all categories of samples that undergo a shift in the same direction during storage. The analysis of the PC scores plot emerging from the multivariate analysis of NMR spectra may help evaluate the quality of a product, particularly when it is necessary to estimate the conformity of a product compared to a standard of quality.

In this specific case study, it is also possible to appreciate the reproducibility of data associated to three different sample preparations of the same fish specimen (replicates). The comparison of such a replicate distance in the PC scores plot with the distance observed among samples of different fish reared in the same aquaculture system provides a way to estimate the stability of quality in the production system. In this light, it is evident that the samples of fish reared in the cage aquaculture system, collected in the present study, have shown a higher variability in quality.

**Figure 3:**
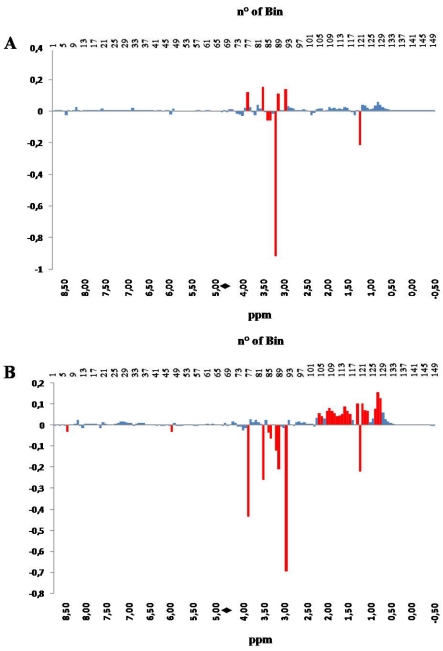
PC1 (panel **A**) and PC 2 (panel **B**) loadings plots corresponding to the PCA performed on binned spectra normalized to constant area. Each bar corresponds to a single spectral bin out of the 150 ones in which the 18,000 spectral data points are grouped. Most relevant bins, or groups of bins, have been colored in red. The black diamond on the ppm scale indicates the omitted solvent (water) bins.

Further details arise from the inspection of the PC loadings plots. PC loadings indicate how much each NMR bin contributes to the description of each PC. In Figure 3, PC1 loadings, panel A, and PC2 loadings, panel B, are shown in a bar-plot, resembling a binned spectrum with positive and negative intensities. The intensity of each bin indicates how much, and in which direction, the spectral variable contribute to the PC.

The inspection of the loading plots indicates that the variations occurring in the hydroxylic region (bins 59–93), collecting signals belonging to amino acids, sugars and hydrophilic compounds, such as taurine, creatine, amines, betaine, *etc.*, have strong influence on PC scores along both PC1 and PC2. On the other hand, changes occurring in the aliphatic region (bins 94–150) have strong influence on the PC2 scores. Differently from the hydroxylic region, which is characterized by the presence of well localized bins affecting the PC2 score, the aliphatic region contributes with a positive correlation as a whole to the value of PC2 score, with the exception of bin 121 (red colored).

This observation is consistent with the fact that a large pool of substances is affected by the application of a specific aquaculture system, suggesting that the evaluation of the quality of fish should be based on a multi-parametric analytical system. The only region having almost no effect on the values of both PC1 and PC2 scores is the aromatic one (bins 1–58), constituted by bins collecting signals belonging to metabolites of the energetic balance (*i.e.*, ATP and its catabolites).

The explanation for this observation is given by considering that all signals present in the downfield region are one order of magnitude less intense, with respect to the signals visible in the other two regions, as a consequence of the fact that the molar fractions of aromatic compounds, including purine derivatives, are much lower than those of lactate, amino acids, sugars and amine (see Table 1).

**Figure 4:**
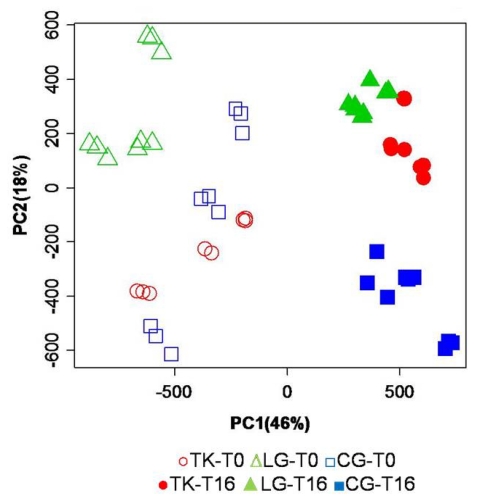
Mean-centered PCA of regionally scaled binned data set, including all samples. The aromatic and the aliphatic regions were vertically expanded 15 and 1.3 fold, respectively, in order to have the same intensity for the highest signal in all three regions. Open symbols refer to samples immediately extracted after fish sacrifice, while filled symbols refer to sample extracted from fish stored under ice for 16 days.

It is worth to note that a non-scaled PCA performed on complex mixtures of a large number of compounds with very different molar fractions is affected more by the absolute amount of each component rather than by their relative variations. In other words, a 5% change in the concentration of a major component determines a larger shift in the PC scores plot than that observed for a 50% change in the amount of a trace compound. This result would underestimate the alteration of the metabolic profile at the level of lower components that are important from a nutritional and/or diagnostic point of view. This occurrence can be partially corrected by performing a second PCA on regionally scaled spectra (Figure 4).

The regionally scaled PCA is performed on spectra that are scaled before multivariate data analysis. In fact, after normalization of the whole spectrum to unitary area, the intensity of all data points in the only aromatic region has been multiplied by 15 fold, in order to have the most intense signals occurring within this region increased to the intensities that are comparable with what observed for signals present in the midfield (hydroxylic) region. The same operation has also been applied to the aliphatic region, but in this case the chosen multiplier is 1.3. In this new PCA, PC1 and PC2 explain 46% and 18%, respectively, of the total variance. The new data transformation reveals a well defined time separation along PC1, where T_0_ samples are located at negative values, while the T_16_ ones are on the positive side. The effect of storage on PC1 scores is amplified because the regional scaling increases the weight of the aromatic region where the signals of the energy related metabolites reside. While along PC1 the clustering is evident due to the effect of storage, the analysis of the second principal component (PC2), explaining 18% of the total variance, is affected by a major contribution from the aquaculture systems. Moreover, this data transformation has amplified the intra-class variance associated to the fish reared in cages, as observed at T_0_, as a consequence of the fact that most of the variability of the composition of fish obtained using this aquaculture system is due to differences in their energetic balance.

**Figure 5:**
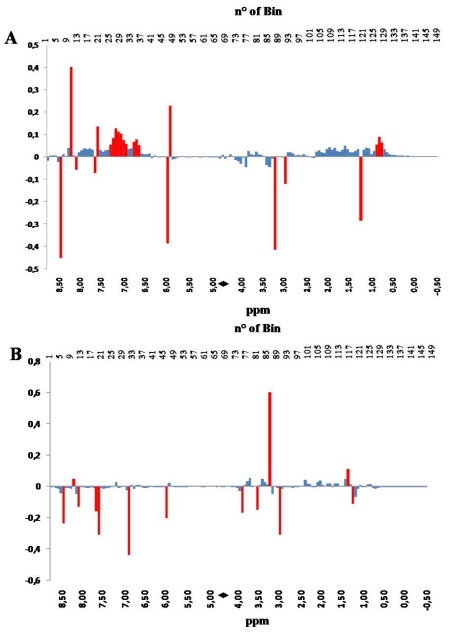
PC1 (panel **A**) and PC 2 (panel **B**) loading plots corresponding to the PCA performed on binned, regionally scaled, spectra. Each bar corresponds to a single spectral bin out of the 150 ones in which the 18,000 spectral data points are grouped. Most relevant bins, or groups of bins, have been colored in red. The black diamond on the ppm scale indicates the omitted solvent (water) bins.

The inspection of the PC loadings bar-plot in Figure 5 confirms what has already been observed with the PCA performed on the original non-scaled data set, consisting of the influence of a broad range of signals present in the aliphatic region of the NMR spectrum, on the separation along the time-dependent PC1 direction. However, in this specific processing of the NMR data, new details emerge from the contribution of the aromatic region of the spectrum, collecting signals from aromatic amino acids and from molecules from the energy and oxidative metabolism, for instance ATP, ADP, INO and IMP (see Table 1). It is evident that the storage moves samples towards higher values of PC1 scores; a shift that is related to the conversion of some metabolites in other compounds. It is worth noting here that the same metabolites contribute to the separation of samples derived from different aquaculture systems along the PC2 scores axis, although another bin (number 32 in Figure 5B, colored in red), not meaningful in PC1, also strongly contributes to that separation.

The overall results of the multivariate data analysis demonstrate that many compounds contribute to the spontaneous grouping of fish samples according to their aquaculture system and, combined altogether, they constitute a couple of condensed parameters, namely PC1 and PC2 scores, still maintaining 64–80% of the total ability to discriminate samples of different quality. In other words, the holistic view is strongly recommended for fish grouping/classification since a broad range of metabolites are involved. Nevertheless, the large information behind the spectral metabolite profile can be condensed into a 2D space, still maintaining most of the original information, represented by a couple of new parameters that are the combination of all of them.

## 4. Conclusions

In this study we have found that metabolomics, the multivariate analysis of metabolic profiles by NMR spectroscopy, represents a powerful tool for detecting differences on a molecular profile basis, that occur in fish as the consequence of storage and the aquaculture system. The chemometric analysis, which the metabolomics approach is based on, shows that fishes at T_16_ exert some statistically significant differences of their metabolic profiles when compared to those at T_0_, providing hints on the time evolution of the fish quality.

As the fish quality is a broad and complex concept embracing many components, which differ in relative importance for producers, processors, marketers, distributors, retailers, caterers, consumers, regulatory authorities and legislators, it is really difficult to establish unique parameters able to define the status of fish quality.

The combination of NMR spectroscopy and an unsupervised chemometric technique for data analysis (PCA) becomes an important tool for detecting differences in the metabolic profiles. The application explored in the present research work may contribute to the definition of a comprehensive descriptor consisting of a pattern of molecular components undergoing significant metabolic changes related to environmental effects, such as the aquaculture systems. 

A comprehensive molecular assignment to signals belonging to bins exerting meaningful variations has not been performed, although we were actually able to identify some, *i.e.*, IMP and its metabolites, as well as some amino acids and glycogen, which turned out to be molecules having a large variance among categories. However, we consciously omitted to exploit this opportunity in order to focus attention on the holistic definition of the molecular quality that can be used for comparison between different origins of the same food product.

NMR spectroscopy, when focused on the identification of biomarkers and key metabolites, makes use of 600 MHz or higher field spectrometers, which provides very good resolution, so that they are considered the standard for metabolomics at a research level. On the other hand, the lower resolution obtained at 400 MHz is good enough for industrial applications and is considered to be an acceptable compromise for obtaining a large amount of information at an affordable cost.

Whatever the spectral resolution reached by the employed instrument, the synergic combination between NMR spectroscopy and chemometrics alone, is not enough for the complete description of quality of fish; particularly when an approach based on water-extract profiles, such as that herein described, is adopted. Nevertheless, the present approach may be successfully used to define, together with other analytical techniques, the point at which the molecular profile of fish is no longer substantially equivalent, from a total quality point of view, to fish of different origin.

The proper strategy for obtaining a robust and accurate measurement of the significant differences among categories is to perform the evaluation on a large set of samples, and extracting predictive models. In this case, metabolomics is also able to find indicators able to predict loss of freshness, as well as to detect differences related to the nutritional status of fish. It still remains to be investigated if this approach may be considered as a criterion for detecting changes of fish quality in a form which is practical both for the fish industry and the consumers.
